# Obesity, Nutrients and the Immune System in the Era of COVID-19

**DOI:** 10.3390/nu13020610

**Published:** 2021-02-13

**Authors:** Jean-Pascal De Bandt, Charlotte Monin

**Affiliations:** Department of Physiology, Faculty of Pharmacy, University of Paris, 75006 Paris, France; charlotte.monin@etu.parisdescartes.fr

**Keywords:** adipocyte, immune cells, leptin, inflammasome, insulin resistance

## Abstract

The past year has shown that obesity is a risk factor for severe complications of SARS-CoV-2 infection. Excess fat mass during obesity is known to be a risk factor for chronic diseases but also for severe infections and infectious complications. We have focused here on the elements responsible for this particular susceptibility to infections and more specifically to COVID-19. Excess fat is, in itself, responsible for alterations of the immune system by disrupting the production and function of immune cells. Indeed, hypertrophic adipocytes produce more pro-inflammatory adipokines (including cytokines). The increase in their apoptosis induces a release of pro-inflammatory compounds into the circulation and a recruitment of pro-inflammatory macrophages into the adipose tissue. A chronic systemic inflammatory state is then observed. In addition, diet, apart from its role in the development of adipose tissue, can also affect the immune system, with excess simple sugars and saturated fats exerting pro-inflammatory effects. This inflammation, the adipokines released by the adipocytes, and the infiltration of lipids into the lymphoid organs affects the production of immune cells and, directly, the functions of these cells. The alteration of the immune system increases the risk of infection as well as complications, including secondary bacterial infections and septic states, and increases infection-related mortality. During COVID-19, the chronic inflammatory state promotes the cytokine shock, characteristic of severe forms, caused in particular by excessive activation of the NLRP3 inflammasome. Furthermore, in obese subjects, the already present endothelial dysfunction will render endothelial inflammation (endotheliitis) due to viral infiltration all the more severe. Added to this is a state of hypercoagulability and a decrease in respiratory capacity, leading to a risk of severe COVID-19 with cardiovascular complications, acute respiratory distress syndrome, and disseminated intravascular coagulation, which can lead to multiple organ failure and even death.

## 1. Introduction

Obesity is a risk factor for many chronic diseases such as type 2 diabetes, cardiovascular disease, and cancer, but also for bacterial, viral, and parasitic infections [1]. Obesity is also responsible for a decrease in the efficacy of vaccines, including rabies, hepatitis B, and influenza vaccines, and a decreased response to antiviral and antibacterial treatments [1].

Recent studies have also shown an association between obesity and the severity of infection with SARS-CoV-2, the causative agent of COVID-19. In obese subjects with COVID-19, an increased risk of severe pneumonia, increased need for mechanical ventilation, increased frequency of complications, and an increased risk of death (acute respiratory distress syndrome (ARDS), disseminated intravascular coagulation (DIC), etc.) and mortality have been observed [2,3]. These observations clearly suggest that obesity has deleterious effects on the immune system, increasing the severity of infections and decreasing the immune system’s ability to produce antibodies.

After some reminders on endocrine activity and adipokine production of white adipose tissue (WAT), we discuss how obesity impacts the innate and adaptive immune systems. Finally, we show how deregulation of the immune system, caused by obesity, can increase the severity of SARS-CoV-2 infections.

## 2. Interactions between Adipose Tissue and Immunity

The endocrine role of WAT is now well established with the secretion of adipokines by adipocytes. Due to the secretion of adipokines such as leptin or adiponectin, WAT is a major player in energy homeostasis not only because of its role in energy storage but also because of its ability to regulate metabolism by communicating with the liver, pancreas, and hypothalamus [4,5]. WAT contains a wide variety of immune cells such as macrophages, neutrophils, mast cells, and B and T lymphocytes [5] which make WAT an immune organ in its own right. Thanks to these immune cells as well as adipocytes, WAT can secrete adipokines with pro-inflammatory properties such as leptin, interleukin-6 (IL6), and tumor necrosis factor α (TNFα), or anti-inflammatory properties such as adiponectin. In this section, we will limit ourselves to leptin and adiponectin, the two main adipokines for physiological regulation of immunity.

### 2.1. Leptin

Leptin is a 16-kDa peptide hormone related to the IL-6 family of cytokines. Leptin receptors are found on a large number of immune cells including cells of innate immunity such as monocytes, macrophages, neutrophils, dendritic cells, natural killer (NK) cells, and of adaptive immunity such as B and T lymphocytes [6]. Leptin acts on the immune system by exerting a pro-inflammatory role. It should be noted that certain cytokines such as TNFα and IL-1 can stimulate its synthesis [6]; leptin secretion is thus increased during infectious and inflammatory processes. It stimulates the inflammatory response during an infection and activates antigen-presenting cells (APCs) [7]. In general, it promotes the pro-inflammatory phenotypes of these cells and their production of pro-inflammatory cytokines, chemotaxis, production of reactive oxygen species (macrophages, neutrophils), cytotoxic activity, or phagocytosis. In natural killer (NK) cells, it increases their cytotoxic activity by increasing the expression of the perforin gene [8], stimulates their secretion of pro-inflammatory cytokines such as IL-6 and TNFα, and protects them from apoptosis [9]. 

At the level of adaptive immunity, leptin promotes the proliferation of CD4+ T lymphocytes and their orientation towards a pro-inflammatory Th1 phenotype with, in particular, an increase in their production of pro-inflammatory cytokines such as interferon (INF)-γ and IL-2 and a decrease in their production of anti-inflammatory Th2 cytokines such as IL-10 and IL-4 [6,8,10]. It promotes the proliferation of Th17 cells but decreases the proliferation of regulatory T cells (Treg) [10]. Leptin increases the proliferation of B lymphocytes, decreases their apoptosis, and activates their secretion of cytokines such as TNFα, IL-6, and IL-10 [9].

### 2.2. Adiponectin

Adiponectin is quantitatively the major adipokine secreted by healthy WAT [11]. In addition to its role in insulin sensitivity, adiponectin also exerts immunomodulatory effects. Its protective effects are well established in the cardiometabolic context [12]; however, it seems to have negative effects in some situations such as rheumatoid arthritis. In macrophages, it promotes the acquisition of the M1 phenotype but with a decrease in the production of pro-inflammatory cytokines (TNFα, IL6, *monocyte chemoattractant protein 1* (MCP1-CCL2)) and an increase in anti-inflammatory cytokines such as IL-10. It also inhibits macrophage apoptosis [8]. The effects on other cells of innate immunity are more complex, both pro- and anti-inflammatory, depending on the molecular form of this adipokine. Adiponectin is also thought to act on adaptive immunity; however, the effects on lymphocytes also seem paradoxical. 

The opposing immunological effects of these two adipokines must be considered in the light of their roles in the regulation of energy metabolism. From a teleological point of view, the balance between pro-inflammatory leptin and anti-inflammatory adiponectin allow, in the healthy individual, the metabolism of energy substrates to be precisely regulated between the maintenance of active cell mass and the protection of the organism against aggressive phenomena, infectious in particular. In the obese patient, the situation changes [12]. Resistance to leptin is observed with a decrease in immune response. The concentration of adiponectin in plasma and WAT decreases while the expression of other adipokines is induced. WAT has been shown to produce a very large number of molecules that are able to interact with different organs via paracrine and endocrine pathways [5], the majority of them having pro-inflammatory properties [4]. Among these molecules, without being exhaustive, we can mention resistin, lipocalin-2, vaspin, omentin, nesfatin, visfatin, retinol binding protein 4 (RPB4), and plasminogen activator inhibitor-1 (PAI-1) [13].

## 3. The Inflammatory State of Adipose Tissue during Obesity

The development of obesity combines diet-related metabolic events, favoring inflammation and insulin resistance, with an energetic imbalance leading to excessive lipid storage; the latter being in turn responsible for the abnormal development of adipose tissue but also for the ectopic deposition of lipids at the origin of inflammatory phenomena. To be able to store more triglycerides, WAT increases its number and size of fat cells. Adipocyte hypertrophy activates endoplasmic reticulum and mitochondrial stress responses, which induce an inflammatory state that becomes chronic [7]. As already mentioned, the development of WAT is associated with a decrease in the secretion of anti-inflammatory adipokines and an increase in the secretion of pro-inflammatory adipokines, notably resistin, TNFα, and IL-6. Finally, a phenomenon of abnormal cell death occurs with the appearance of apoptotic adipocytes [8].

### 3.1. Obesity and Inflammasome

Although inflammatory phenomena have traditionally been described in the context of the immune response to exogenous factors, numerous studies over the last two decades have highlighted the capacity of endogenous stressors, particularly metabolic ones, to induce inflammatory phenomena. Briefly, in the context of the innate response to exogenous pathogens, pathogen-associated molecular patterns or PAMPs are recognized by immune cells due to pattern recognition receptors (PRR), which include membrane receptors such as Toll-like receptors (TLR) or intracellular receptors such as NOD receptors. Work at the beginning of this century showed that endogenous molecules, the alarmins or damage-associated molecular patterns (DAMP), were able to activate the same pro-inflammatory pathways. These DAMPs can be proteins released during cellular stress such as high-mobility group protein B1 (HMGB1), S100 proteins, or certain heat shock proteins, or substances such as uric acid or cholesterol crystals. PAMP and DAMP can interact directly with PRRs or activate, indirectly via oxidative stress, inflammasomes. The latter, and in particular the inflammasome nucleotide-binding and oligomerization domain (NOD)-like receptor, leucine-rich repeat, and pyrin domain-containing 3 (NLRP3), are intracellular protein complexes present in macrophages but also in the cytoplasm of dendritic cells, neutrophils, and B and T lymphocytes [14] or endothelial cells. These inflammasomes are normally activated during infections with pathogens whose PAMPs are recognized by PRRs such as TLR4 which recognizes, among others, lipopolysaccharides (LPS). Inflammasome activation requires two different signals: One involving activation of the expression of inflammation genes, for example via the nuclear transcription factor-kappa B (NF-kB) pathway, and the other involving phenomena such as oxidative stress or mitochondrial stress [14,15]. Once activated, inflammasomes cleave pro-caspase 1 into caspase 1 which in turn allows the release of the active forms of IL1 and IL-18. In addition, caspase 1 is responsible for the cleavage of gasdermin D and the formation of a membrane pore leading to inflammatory cell death or pyroptosis and the associated release of inflammatory components [16,17]. IL-1 is a potent pro-inflammatory cytokine that is finely regulated by the inflammasome and recruits other pro-inflammatory cells at the site of infection. IL-18 induces the secretion of TNF-α, IL-1, and IL-8 and induces the differentiation of T lymphocytes into pro-inflammatory Th1 cells [18]. Activation of the inflammasome cascade during an infection normally stimulates an adaptive innate response that promotes its resolution [19].

In obese subjects, there is an increase in the expression of the NLRP3 inflammasome [3] and several factors can contribute to its activation, beginning with metabolic factors.

### 3.2. Diet and Inflammation

Immune disorders are pre-existing even before obesity is constituted, as shown by the presence of a chronic low-grade inflammatory state in the metabolic syndrome, a syndrome associating to varying degrees visceral accumulation of fat, hypertension, non-alcoholic fatty liver disease (NAFLD), glucose intolerance, and dyslipidemia with hypertriglyceridemia and decreased HDL-cholesterol [20]. It is sometimes difficult to distinguish between causes and consequences, especially since some nutrients contribute to the development of inflammatory phenomena while inflammation, particularly through insulin resistance, contributes to metabolic disorders. Independent of obesity, nutrients can influence the state of activation of the inflammasome and thus the development of an inflammatory state. The activity of the NLRP3 inflammasome can be influenced by many factors, starting with nutritional status. It is, for example, stimulated in the post-prandial phase and reduced by fasting through mechanisms dependent on cellular energy and sirtuin 3 [14].

The pro-inflammatory factors associated with diet begin in the gastrointestinal tract with possible imbalances of the microbiota and alterations in the integrity of the intestinal barrier. The most striking example is the translocation of LPS favored by a high-fat diet [21]. Conversely, in experimental models of high fructose diet, the induction of NAFLD can be prevented by antibiotics [22]. In general, these interactions between some nutrients, the microbiota and bacterial translocation seem to be largely involved in the development of NAFLD [23]. In addition, the diet, especially high fat diets, and insulin resistance can lead to a decrease in the production of the mucus that protects the intestinal epithelium, epithelial stress, and changes in the expression of epithelial intercellular junction proteins with increased intestinal permeability [24,25,26], promoting the translocation of bacterial components such as endotoxin [27,28,29].

While the relationship between simple carbohydrate intake and triglyceridemia has long been known, various studies in recent years have highlighted the pro-inflammatory effect of too frequent fluctuations in blood glucose levels compared to a constantly high blood glucose level, and have thus underlined the pro-inflammatory influence of foods with a high glycemic index. Thus, intermittent hyperglycemia is experimentally associated with an activation of myeloid lineages in the bone marrow, an increase in circulating pro-inflammatory monocytes and neutrophils [30,31], an increase in plasma concentrations of pro-inflammatory cytokines associated with increased oxidative stress, and endothelial dysfunction [32]. In vitro, intermittent exposure of macrophages to high glucose concentrations is responsible for a M1 polarization of these cells [33] by mechanisms associated with the activation of TLR4 and c-Jun N-terminal kinase (JNK) [34].

Different substrates such as saturated fatty acids or omega-3 fatty acids may also have activating or inhibiting effects on the inflammasome, respectively [14]. Saturated fatty acids such as palmitate via TLR4-associated mechanisms can activate the NLRP3 inflammasome [35,36,37]. Several mechanisms may be involved: Direct activation via TLR4 and PRR, oxidative stress, or endoplasmic reticulum stress via its protein kinase R (PKR)-like endoplasmic reticulum kinase (PERK) and inositol-requiring enzyme 1 (IRE1) pathways [14,35,38,39,40].

More globally, the various modifications of the plasma lipid profile will participate in this activation of the inflammasome and thus in the inflammatory state: The increase in the mass of adipose tissue and thus in the plasma concentrations of free fatty acids, the decrease in HDL cholesterol which normally contributes to neutralizing bacterial toxins such as LPS [41], and the imbalance in LDL composition in favor of small dense lipoproteins that are very sensitive to oxidation and promote inflammation when trapped by macrophages [42]. 

It should be noted that these interactions between diet, intestinal function, low-grade inflammation, and morbidity, particularly infectious morbidity, are also widely evoked in the development of aging-associated disorder and “inflammageing” [43].

### 3.3. Pro-Inflammatory Adipokines

Hypertrophic adipocytes secrete pro-inflammatory cytokines such as TNFα and IL-6. Their production is increased in cases of obesity but also in the event of inflammatory stimulation by bacterial toxins such as LPS [5]. These two cytokines contribute to the decrease in insulin sensitivity observed in obesity and metabolic syndrome. TNFα decreases insulin sensitivity indirectly by increasing the concentration of free fatty acids (FFAs) in the circulation but also by directly blocking the transmission of insulin signals. It blocks glucose uptake by cells by inhibiting the expression of the GLUT-4 glucose transporter gene. As for IL-6, it increases insulin resistance but also increases the hepatic production of acute phase proteins such as C-reactive protein (CRP), fibrinogen, and haptoglobin, which are systemic markers of acute inflammation [44].

Hypertrophic adipocytes also produce resistin, a pro-inflammatory adipokine. The transcription of its gene is induced by pro-inflammatory cytokines such as IL-1, IL-6, and TNFα. In turn, resistin increases the expression of TNFα and IL-6 and increases the expression of vascular cell adhesion molecule 1 (VCAM1) and intercellular adhesion molecule 1 (ICAM1), two molecules present on the surface of the vascular endothelium and circulating immune cells such as lymphocytes, monocytes, and eosinophils, and allowing the latter to adhere to the endothelium and then pass into the underlying tissue. Resistin also increases insulin resistance and reduces glucose tolerance.

### 3.4. Adipose Cell Apoptosis

The persistence of stress and inflammation in the WAT will lead to hypoxia of the adipocytes and an increase in their apoptosis. Obese patients present crown-like structures in WAT which are apoptotic adipocytes surrounded by macrophages. These structures are found in large numbers and are associated with increased concentrations of pro-inflammatory adipokines. They reflect on the one hand the pro-inflammatory state and on the other hand the deficient phagocytic activity of the macrophages [45].

Apoptosis of adipocytes leads to the release of chemotactic mediators such as MCP-1, which allows infiltration of WAT by macrophages. In healthy WAT, macrophages represent 5–10% of the cells, compared to 50% of the cells in obese patients [41]. These macrophages express a pro-inflammatory M1 phenotype instead of the anti-inflammatory M2 phenotype expressed in healthy tissue [9]. This is promoted by the increased production of pro-inflammatory cytokines and decreased number of eosinophils which normally promotes macrophage M2 anti-inflammatory phenotype via IL4 secretion [10,46]. M1 macrophages will in turn secrete pro-inflammatory cytokines such as IL-6 and TNFα, but also MCP-1 which enhances infiltration by the macrophages [4]. In addition, WAT pre-adipocytes have the ability to transform into macrophages under the effect of these inflammatory signals [47].

There is thus on the one hand a secretion of pro-inflammatory cytokines by macrophages that infiltrate the WAT and on the other hand an increase in the secretion of pro-inflammatory adipokines by adipocytes. These pro-inflammatory cytokines in turn activate immune signaling pathways [47]. Obesity thus leads to a chronic inflammatory state of WAT as well as to a systemic inflammatory state.

## 4. Dysimmunity of Obesity

The immune system is composed of two parts: The innate immune system and the adaptive system. They depend first on the proper differentiation of the various cell populations in lymphoid tissues such as bone marrow or thymus. It also depends on the coordination between innate and adaptive responses [41,48]. The coordination of these two systems allows for the elimination of pathogens via pro-inflammatory processes as well as the production of memory cells that allow a faster immune response during reinfection [48]. The immune system must be able to activate the inflammatory process but also to resolve it when the pathogen is eliminated. This therefore also implies the subsequent suppression of inflammatory phenomena, activation of anti-inflammatory cells, tissue repair, and return to normal. 

Obesity and metabolic syndrome are characterized by an inability to resolve inflammatory phenomena. The chronic inflammation of WAT found in obesity and the resulting abundance of pro-inflammatory mediators in WAT are associated with metabolic dysfunctions such as hyperlipidemia, hyperglycemia, and insulin resistance that will directly and indirectly affect immune organs and cells [41]. Mediators such as MCP1, TNFα, and IL-6 increase lipolysis and decrease triglyceride synthesis. As a result, there is an increase in the release of FFAs into the bloodstream and their accumulation in the liver, skeletal muscle, and β cells of the pancreas [44], with a number of FFAs also having pro-inflammatory properties.

### 4.1. The Consequences of Obesity on Lymphoid Tissues

The WAT of obese subjects releases FFAs into the bloodstream that will accumulate in metabolic tissues but also in primary lymphoid organs such as bone marrow and thymus [41]. The bone marrow is an organ that produces and allows the differentiation, maturation, and proliferation of hematopoietic stem cells from which immune cells of the lymphoid (B and T lymphocytes) and myeloid (monocytes/macrophages, dendritic cells, granulocytes, and mast cells) lines are derived. For its part, the thymus allows the maturation of T lymphocytes. The integrity of these immune tissues is essential for the production and differentiation of the different immune cells. Increased adiposity in primary lymphoid tissues alters their integrity and immune cell production [41].

In bone marrow, adipocytes play an important role in immunity. They secrete survival factors for cells of adaptive immunity and allow for the establishment of nests for memory lymphocytes. Increased adiposity in bone marrow has a deleterious effect. Indeed, in obese subjects a decrease in hematopoiesis is observed with a decrease in hematopoietic stem cells and a decrease in the proliferation of lymphoid progenitors but an increase in myeloid progenitors [40]. In addition, there is an increase in pro-inflammatory gene expression with increased production of IL-15, IL-6, INFγ, TNFα, and ROS [1,13].

In the thymus, the accumulation of adipocytes alters its architecture [41]; its integrity is important because it allows T lymphocyte maturation and prevents T lymphocyte dysfunction and self-reactivity. In obese subjects, a reduction in T lymphocyte precursors is observed with a decrease in the production of naïve T cells and T cell-dependent immune monitoring [20].

Obesity also impacts secondary lymphoid tissues: The spleen, lymph nodes, and mucosa-associated lymphoid tissue (MALT). In the spleen of obese subjects, there is an increase in the effector-to-memory T cell ratio and a decrease in T cell receptor (TCR) diversity. Obesity therefore reduces the repertoire of circulating T cells and the repertoire of antigens to which they can respond. There is also a decrease in the size of the inguinal lymph nodes, a decrease in the number of circulating T cells, as well as a decrease in lymphatic circulation, which decreases the migration of dendritic cells to the peripheral lymph nodes [49].

### 4.2. The Consequences of Obesity on Innate Immunity

The innate immune system consists of myeloid cells including monocytes/macrophages, dendritic cells, mast cells, NK cells, and granulocytes (neutrophils, basophils, eosinophils). It is the first line of defense in case of infection or injury [41].

#### 4.2.1. Neutrophils

Neutrophils limit the growth or even eradicate pathogens due to their antimicrobial activity based on the increased expression of myeloperoxidases, and the production of proteins such as defensins and cathepsin G contained in the granules of the cytoplasm of these cells [50]. 

Obesity disrupts the function of neutrophils. Indeed, an elevation in the basal state of the production of a ROS, the superoxide anion, is observed. The neutrophils of obese subjects are therefore already activated in the basal state [21]. On the other hand, their response to an infectious stimulus is decreased compared to that of non-obese subjects: When neutrophils are activated by an infectious stimulus, e.g., LPS of Gram-negative bacteria, there is a decrease in neutrophil chemotaxis in response to keratinocyte chemoattractant (KC or IL-8) and formyl-methionyl-leucyl-phenylalanine (fMLP), as well as a decrease in their production of pro-inflammatory cytokines such as IL-1β, IL-6, TNFα, and MCP1 [51]. Thus, in obesity, there is an increase in oxidative stress and the chronic basal inflammatory state [50] and a decrease in the host response to infections, as neutrophils cannot contain bacterial growth [51].

#### 4.2.2. Macrophages

Macrophages are tissue immune cells derived from blood monocytes. They have a phagocytosis role, i.e., they eliminate apoptotic or necrotic cells and pathogens. They also act as antigen-presenting cells: They present antigen to naive T lymphocytes and activate them via the major histocompatibility class 2 complex (MHCII). These are the most abundant immune cells in adipose tissue [10].

We have previously seen that WAT macrophages in obese patients express a pro-inflammatory M1 phenotype and secrete pro-inflammatory cytokines such as TNF-α, IL-6, and IL-1β. Adiponectin deficiency contributes to this shift of macrophages from the anti-inflammatory M2 phenotype to the pro-inflammatory M1 phenotype. Thus, in the basal state, there is a chronic polarization into pro-inflammatory M1 macrophages [49]. Through chemokine secretion, these M1 macrophages will attract peripheral monocytes which will in turn differentiate into M1 macrophages [49]. It can be noted that already in the basal state the circulating monocytes of obese patients have increased secretion of pro-inflammatory cytokines [52]. On the other hand, these macrophages show a decrease in phagocytic function illustrated experimentally, for example, by a decrease in the clearance of *Escherichia coli*, *Candida albicans*, or *Klebsiella pneumoniae* and an increase in mortality in ob/ob mice [53]. Correcting the M1/M2 ratio of macrophages improves their function [10].

#### 4.2.3. Dendritic Cells

Dendritic cells are the sentinels of the immune system. They recognize infectious signals and initiate the activation and polarization of naïve T lymphocytes by acting as antigen-presenting cells. They stimulate the differentiation of T lymphocytes into pro-inflammatory Th1 or immune-modulatory Th2 [48,54]. 

In obese subjects, the number of circulating dendritic cells is decreased in the basal state and after stimulation [54,55]. However, because of the chronic inflammatory state, the number of inflammatory dendritic cells increases in the WAT; these cells, which are not normally present in the basal state, contribute to inflammation of the WAT and to the secretion of IL-17 by pro-inflammatory Th17 T cells [48]. During infectious episodes, increased apoptosis of dendritic cells is observed in obese subjects. In addition, the cytokine response of dendritic cells to antigens is deregulated with an increase in the production of IL-10 and IL-4, two immune-modulatory cytokines: IL-4 induces the differentiation of T lymphocytes into immune-modulatory Th2 cells [54]. This deregulation therefore decreases the efficiency of the T lymphocyte response and increases the susceptibility of obese subjects to viral infections.

#### 4.2.4. NK Cells

NK cells are cytotoxic lymphocytes which, unlike cytotoxic CD8+ T lymphocytes, do not require prior activation by an antigen to destroy their target cells which may be tumor cells or cells infected by pathogens. They exert their cytotoxic properties through the production of cytotoxic molecules such as granzymes and perforins, through the activation of apoptosis by death ligands such as TNF-related apoptosis-inducing ligand (TRAIL), or by antibody-dependent cytotoxicity. Once activated they also produce pro-inflammatory cytokines such as INFγ and TNFα which will recruit the adaptive system [55,56]. 

In obesity, there is an increase in the number of NK cells in adipose tissue, mainly due to local proliferation of these cells, especially in visceral adipose tissue. There is an increase in the NK cell activating receptor ligand on adipocytes, which induces NK cell proliferation and the production of IL-6, TNF, and INFγ [56]. This favors the differentiation of M1 macrophages and an increase in WAT inflammation. On the other hand, the number of circulating NK cells, their capacity to produce anti-inflammatory cytokines, and their cytotoxic function are also decreased [10,51,56], which increases mortality in sepsis and the risk of cancer development. 

### 4.3. The Consequences of Obesity on Adaptive Immunity

The adaptive system amplifies the immune response and confers a specific response to a given pathogen as well as a memory response due to the production of memory lymphocytes that allow a faster and more efficient response in case of reinfection by the same pathogen. Activation of the adaptive system depends on the specific interaction with cells of the innate system involving antigen presentation by antigen-presenting cells and activation of lymphocytes [41].

#### 4.3.1. T Lymphocytes

T cells are responsible for cellular adaptive immunity, the role of which is to specifically eliminate virus-infected cells or cancer cells after activation by an antigen-presenting cell. Different cell populations and sub-populations must be considered. Tγδ cells can contribute to both innate and adaptive immunity and produce pro-inflammatory cytokines and lytic (perforin) and apoptotic factors (TNFα, TNF-related apoptosis-inducing ligand (TRAIL), apoptosis stimulating fragment receptor ligand (FasL)). They play a particular role in viral infections, e.g., influenza A virus [57]. The cytotoxic CD8+ T cells eliminate by lysis (perforin) or apoptosis (granzyme and other serine protease) the cells presenting their specific antigen. CD4+ T cells are necessary intermediaries for the activation of other cells. After activation by MHCII, they differentiate into several subtypes: Th1 that secretes INFγ and IL2 (required for activation and amplification of the B cells), Th2 that secretes IL-4, IL-5, and IL-13, Th17 that secretes IL17, and Treg that has an immunosuppressive action on other LTs by secreting anti-inflammatory cytokines such as IL-10 or TGF-β, by binding IL-2, or by having a direct cytotoxic action.

In obese subjects, there is a decrease in the global T cell population and in their activation and function [58]. The number of Tγδ cells and their proliferation are decreased, and the remaining cells produce less IFNγ which decreases their antiviral activity. This induces persistence of infection and increases mortality during sepsis [48]. The number and function of CD4+ and CD8+ effector T cells are also decreased. For example, when stimulated by the H1N1 virus, effector T cells in obese subjects produce less INFγ, Granzyme B, TNFα, and IL-6, which alters bacterial clearance and allows the infection to persist. Paradoxically, the number of CD4+ and CD8+ T cells increases in visceral WAT where CD4+ T cells differentiate into Th17 and Th22 and produce pro-inflammatory cytokines which increase local hyperinflammation [48,58]. As for Treg, their number decreases in WAT which induces a decrease in the suppression of effectors T cells and reinforces inflammation [10]. The resolution time of the infection and the risk of complications are thus increased. Note that the exact influence of obesity on memory T cells and on secondary infections remains to be evaluated [59].

This loss of function of CD4+ and CD8+ effector T cells as well as the decreased production of memory T cells are partly related to the deregulation of the metabolism of these cells. In their quiescent state, naïve T cells use oxidative phosphorylation via mitochondrial respiration to provide energy for immune monitoring and homeostasis [10]. During an infection, naïve T cells are activated and need to rapidly differentiate, proliferate, and secrete cytokines. For this, they need a rapid increase in ATP production but also the availability of substrates for cell growth and response. As a result of TCR activation, T cells undergo a change in their metabolism marked by an increased uptake of glucose and glutamine and their incomplete oxidation in anaerobic glycolysis and glutaminolysis to provide the necessary reaction intermediates for the synthesis of lipids, proteins, and nucleotides to support cell growth and proliferation [10]. Leptin plays an essential role in this activation of T cells because it activates this glucose metabolism. However, in obese subjects, there is resistance to leptin, which leads to a decrease in the uptake and use of glucose, an effect reinforced by insulin resistance [60]. There is therefore a decrease in the differentiation of naïve T cells into effector cells. Note that these metabolic disturbances also affect the formation and maintenance of memory T cells [60]. This decrease results in an increase in mortality from secondary pathogen infection, as well as a failure to acquire immune protection after vaccination [60].

#### 4.3.2. B Lymphocytes

Among the B cells, a majority differentiate, after activation, into plasmocytes secreting antibodies that neutralize the antigens or facilitate their elimination, for example by recruiting cytolytic cells. Some B cells differentiate after activation into memory cells. Finally, B cells also produce pro-inflammatory and anti-inflammatory cytokines depending on their degree of maturation.

In obesity, defective humoral immune response has been described [61,62]. The activation-induced deaminase (AID), which allows somatic hypermutation of B cells, is decreased; therefore, there is a decrease in the production of antibodies. There is also a decrease in memory B cells as well as a decrease in their function, which induces a decrease in the response to infection but also a decrease in the vaccine response [62]. Finally, there is an increase in the production of pro-inflammatory cytokines by B cells in the basal state [10,48]. These cells show an increase in the expression of Toll-like receptor 4 (TLR4) (which, via activation of NF-kB, increases the secretion of IL-6, IL-1β, TNFα, and MCP1 in response to LPS or certain FFAs) and a decrease in the production of anti-inflammatory cytokines such as IL10 [62]. These cells will also promote the inflammatory phenotype of T lymphocytes [10] by stimulating their production of IL-17 and IFN-γ [62]. B lymphocytes in obese subjects therefore express a pro-inflammatory phenotype in the basal state that decreases their capacity to generate an optimal response to an infection. In addition, pro-inflammatory B lymphocytes also infiltrate visceral adipose tissue and promote its inflammation and insulin resistance [48]. 

## 5. Obesity and COVID-19

### 5.1. Coronavirus Disease-2019

The COVID-19 pandemic, which began in November 2019 in China, has resulted in the deaths of more than 1.62 million people worldwide as of 15 December 2020. This pandemic is caused by the severe acute respiratory syndrome coronavirus 2 (SARS-CoV-2) virus, which belongs to the Coronaviridae family, characterized by a crown-shaped viral envelope. This virus has a mode of human-to-human transmission via the respiratory tract via microdroplets. It targets the angiotensin-converting enzyme 2 (ACE2) which is found in many tissues, particularly in respiratory cells, vascular endothelial cells, and myocardial cells. Once the virus enters the cell, it causes apoptosis of the target cell, triggering activation of the production of pro-inflammatory cytokines and chemokines and the recruitment of inflammatory cells [2], probably in part via the involvement of the NLRP3 inflammasome [15,63]. The virus also increases the apoptosis of CD3, CD4, and CD8 T cells, which induces lymphopenia and impaired lymphocyte function. Hypercytokinemia or cytokine shock is responsible for the severe complications of SARS-CoV-2 infection [3].

COVID-19 symptoms are varied. Patients may be asymptomatic, have flu-like symptoms with fever, dry cough, and fatigue, but may also have respiratory symptoms that can progress to ARDS, which is manifested by dyspnea, hypoxemia, respiratory failure, and abnormalities visible on thoracic imaging. ARDS requires hospitalization in the intensive care unit and mechanical ventilation [3]. COVID-19 may cause secondary infectious complications, cardiovascular disease such as myocarditis, arrhythmia, myocardial infarction, or stroke, and clotting disorders such as thrombosis or DIC.

### 5.2. Obesity: A Risk of Severe COVID-19

Malnutrition, whether undernutrition or obesity, is linked to a poor prognosis for viral infections, notably due to a defective innate and adaptive immune response. It was observed during the H1N1 pandemic in 2009 that obesity increased the severity and mortality of infection and the risk of hospitalization [64]. Because of the similarities between the influenza virus and the SARS-CoV-2 virus, studies have been conducted to determine the link between obesity and the severity of COVID-19. Very soon after the outbreak of the epidemic, Chinese data showed that overweight and obese people had an 86% and 142% increased risk, respectively, of developing pneumonia secondary to COVID-19 [65]. Obese people are indeed more at risk of developing severe forms of the disease as shown in a French study in which 75.8% of patients with a severe form of COVID-19 had a BMI > 30 kg/m^2^ [3] with an odds ratio for invasive mechanical ventilation of 7.36 in patients with BMI > 35 kg/m^2^ vs. BMI < 25 kg/m^2^. Obese patients are therefore more at risk of being hospitalized in intensive care units [64] and requiring mechanical ventilation [2]. 

Several mechanisms may contribute to this severe expression of COVID-19. First, there is an increase in the expression of ACE2, the target of the SARS-CoV-2 virus, in WAT and lung tissue in obese subjects and insulin resistance may contribute [2]. This viral tropism for these two tissues may promote invasion and proliferation of the virus. In addition, we have previously seen that obesity is associated with a chronic inflammatory state and an increase in the production of pro-inflammatory cytokines and adipokines that induce deregulation of the innate and adaptive immune response during an infectious process. This represents a breeding ground for the hyper-inflammatory response and cytokine shock in severe cases of COVID-19 [3].

#### 5.2.1. Obesity, COVID-19, and Hyperinflammation

In obese subjects, the increased expression of the NLRP3 inflammasome is, during the course of infection with SARS-CoV-2, additive to the activation of the inflammasome by the virus and leads to overactivation of the inflammasome and hyperproduction of inflammatory cytokines. This increases the dysfunction of adaptive immunity with a decrease in B and T lymphocytes and their functions. The resulting hypercytokinemia induces cytokine shock, particularly in the lungs, which is the cause of ARDS in severe forms of COVID-19. It should be noted that, by their very nature, obese subjects are already at greater risk of ARDS because they often present pulmonary disorders such as obstructive bronchopathies with an increase in airway resistance, a decrease in the strength of respiratory muscles, and therefore an insufficient compensatory respiratory response in the event of pulmonary infection. Subsequently, ARDS can lead to multiple organ failure, respiratory arrest, and death [2].

#### 5.2.2. Obesity, COVID-19, and Endothelial Dysfunction

Patients with severe forms of COVID-19 also have cardiovascular and thrombotic events such as infarction, myocarditis, thromboembolism, and DIC.

Obesity itself is frequently associated with the co-morbidities of the metabolic syndrome and therefore with an increased risk of cardiovascular diseases. Obesity is an independent risk factor for coronary heart disease; it is also a risk factor for the development of heart failure as a consequence of its deleterious effects on left ventricular function [3]. The chronic inflammation that occurs in obese people induces an increase in coagulation factors such as tissue factor (TF) or PAI-1, as well as a decrease in tissue factor pathway inhibitor (TFPI), antithrombin, and protein C. Obesity is also associated with endothelial dysfunction, which may contribute to the severity of the disease. Indeed, insulin resistance, hyperinsulinemia, and leptin resistance induce a decrease in the expression of nitric oxide synthase (NOS) in endothelial cells. This results in lower nitric oxide (NO) production and thus a reduction in its anti-inflammatory and vasodilatory effects [3]. Leptin resistance also induces the production of MCP-1 which leads to leukocyte infiltration of the vascular walls [3]. In addition, metabolic syndrome dyslipidemia promotes oxidative stress in the vascular wall which contributes to further decreasing the availability of NO and enhances local macrophage activation [3].

The already fragile cardiovascular function of the obese patient is exposed to an additional risk because ACE2, the target of SARS-COV-2, is present on endothelial cells. A post-mortem histological study on 3 patients showed the presence of viral inclusions in endothelial cells, an accumulation of pro-inflammatory cells, as well as apoptotic bodies in the endothelial cells of the heart, small intestine, and lungs [19]. This suggests that the virus is responsible, directly or through the recruitment of pro-inflammatory cells, for endotheliitis, a specific inflammation of the vascular endothelium, and causes endothelial dysfunction in susceptible subjects [19].

In obese subjects, for whom endothelial dysfunction is pre-existing at the time of infection, the clinical presentation of endotheliitis is more severe and may lead to cardiovascular complications, ARDS, and a state of hypercoagulability favoring thrombosis. All this may lead to multiple organ failure and death [3].

## 6. Conclusions

At the peak of the first wave of SARS-CoV-2 contamination, obesity rapidly emerged as a major risk factor for mortality during this infection and the reasons for this are both immune and metabolic.

In obesity, AT dysfunction leads to an increase in the secretion of pro-inflammatory adipokines and a chronic inflammatory state first of all of adipose tissue but becoming systemic through the release of pro-inflammatory adipokines and cytokines into the circulation. The chronic inflammatory state of obesity directly impacts innate and adaptive immunity cells with both a pro-inflammatory orientation of immune cells in the basal state and results in a decrease in their ability to respond to infection and re-infection. In addition, metabolic disorders affect the integrity of lymphoid organs and their production of immune cells, but also the ability of immune cells to adapt their metabolism to respond to inflammatory phenomena. In obese patients, the situation is further weakened by the frequent occurrence of cardiorespiratory abnormalities and more particularly endothelial dysfunction.

In COVID-19, increased expression of ACE2 in obese subjects contributes to activation of the NLRP3 inflammasome and hyperproduction of pro-inflammatory cytokines that are superimposed on the chronic inflammatory state to promote the cytokine shock characteristic of severe COVID. Endotheliitis caused by viral infection added to vascular dysfunction then leads to cardiovascular complications, thrombosis, ARDS, multiple organ failure, and death.
